# Quantification of the relative contribution of phase aberration and reverberation in transcranial ultrasound imaging: an experimentally calibrated fullwave study in 2D and 3D

**DOI:** 10.1088/1361-6560/adf2f3

**Published:** 2025-08-11

**Authors:** Danai E Soulioti, Rebecca M Jones, Gianmarco F Pinton

**Affiliations:** Lampe Joint Department of Biomedical Engineering, University of North Carolina at Chapel Hill and North Carolina State University, Chapel Hill, NC 27599, United States of America

**Keywords:** aberration, fullwave simulations, reverberation, transcranial, ultrasound imaging, ultrasound simulations

## Abstract

*Objective.* The skull significantly aberrates ultrasound imaging pulses due to its acoustic properties and morphology. However, in addition to aberration of sound waves, the large speed of sound and density mismatch between soft tissue and bone is responsible for multiple reverberations between tissue interfaces and the transducer. Even though a significant amount of research has been dedicated to measuring, characterizing, and correcting the phase aberration caused by the skull, comparatively few results exist on multiple reverberation. The objective of this paper is to quantify reverberation clutter in the brain and to compare degradation from clutter and aberration. *Approach.* A full-wave equation simulating nonlinear propagation in a heterogeneous medium is solved numerically to explore the degrading effects of the human skull. Simulations were performed using isovelocity and clutter subtraction simulations to compare the relative contributions of reverberation and aberration on point spread function degradation. *Main results.* From the performed simulations, it is shown that (a) reverberation is significant in transcranial imaging due to the inclusion of both transmit and receive pulses during imaging, (b) the effect of aberration on image degradation is independent of target brightness whereas the effect of reverberation is dependent on target brightness, (c) reverberation is depth dependent, and (d) the microstructure has little impact on overall reverberation properties in thin skull regions. *Significance.* From this study, it shown that to further improve transcranial ultrasound imaging, especially with respect to lower amplitude and shallower targets, both aberration and reverberation should be addressed.

## 1. Introduction

Research on transcranial ultrasound propagation has been directed towards therapeutic applications (Hynynen and Jolesz 1998, Tanter *et al* 1998, Aubry *et al* 2003), and to a lesser extent, towards brain imaging. Transcranial imaging has been mostly explored in the context of vascular brain imaging using transcranial Doppler sonography (Aaslid *et al* 1982), with the ability to monitor blood velocity in a real-time color-coded manner (Bogdahn *et al* 1990). The degradative effects of the skull on transmission energy have long been known in brain imaging and therapy (Grolimund 1986, Fink 1992), where the skull is a strong phase aberrator that degrades the image quality. Several methods have been developed in the past decades to correct this aberration, restoring the ultrasonic beam using a time-reversal based approach or cross-correlation based methods (Tanter *et al* 1998, Ivancevich *et al* 2006). However, multiple reverberations, within the skull and between the skull and the transducer, also have an impact on image quality, yet their effect has not been extensively studied or characterized. The objective of this paper is to quantify the reverberation clutter in brain imaging, to determine its effect on image quality, and to determine its contribution to image degradation relative to phase aberration.

**Figure 1.**
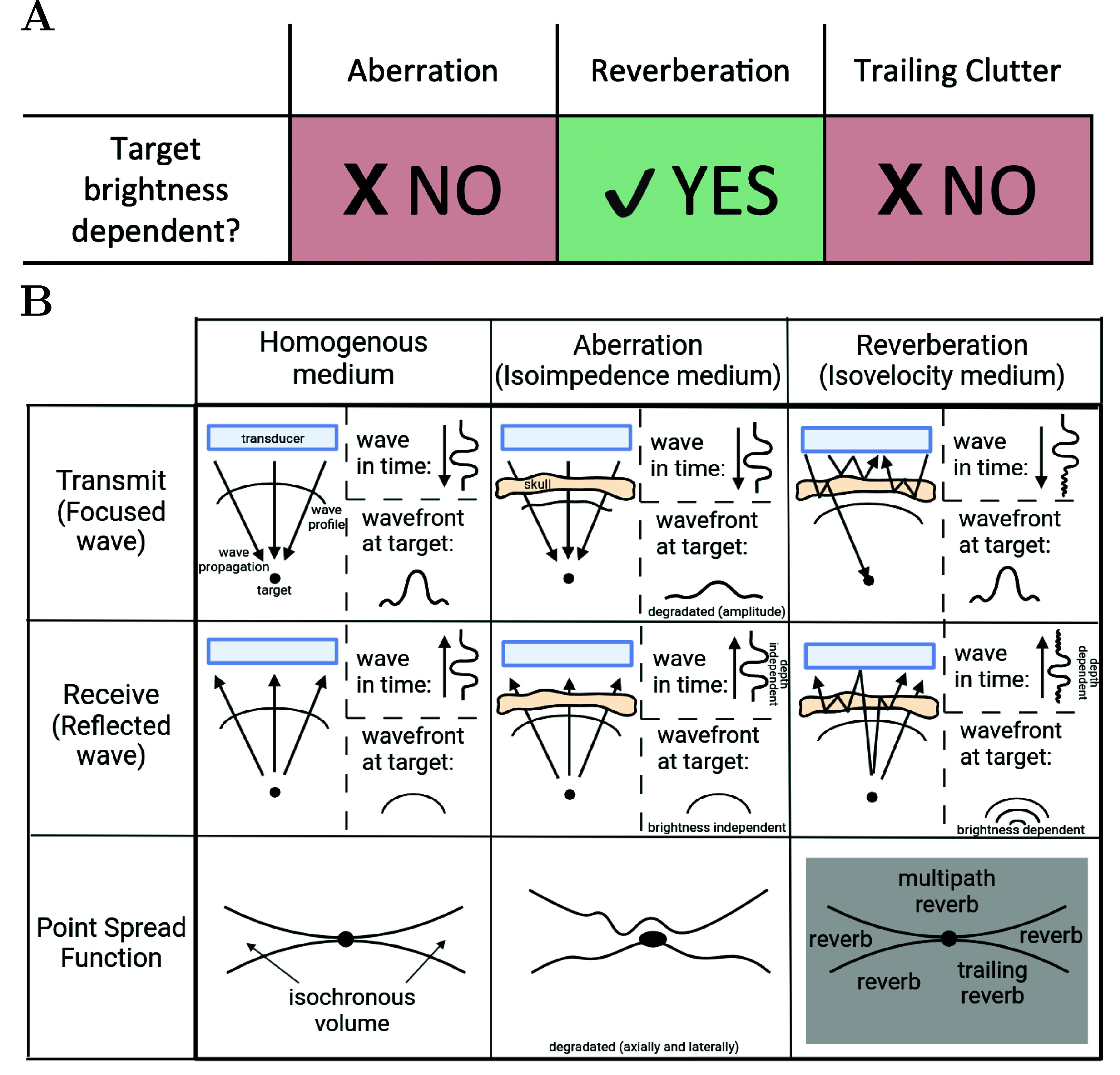
(A), Representation of the difference between aberration, reverberation, and trailing clutter in target brightness dependency, where reverberation is target brightness dependent and aberration and trailing clutter are not. (B), Schematic illustrating the effects of multiple reverberations and aberration on a PSF during transcranial imaging. Homogeneous case provided as a reference.

Figure 1(A) demonstrates the difference in target brightness dependency between aberration, reverberation, and trailing clutter, where reverberation is target brightness dependent. A more detailed explanation is shown in figure 1(B), which illustrates schematically the effects of reverberations and aberration on the point spread function (PSF) generated from a point scatterer in a homogeneous medium (1st column) as well as in the absence of reverberations (2nd column) and aberration (3rd column). Two sources of image degradation are the most prevalent: phase aberration, stemming from the heterogeneous composition of the skull that distorts the phase and amplitude of the wave, and reverberation, caused by the multiple reflections in the heterogeneous layers of the skull. Phase aberration can be calculated using a isoimpedance medium, or a medium with a constant impedance value, and multiple reverberations can be calculated using a isovelocity medium, or a medium with constant speed of sound (Pinton *et al* 2011c). The PSF is described by the ‘X’ shaped region at the depth of the scatterer and is illustrated in the bottom row of figure 1(B). The PSF can be divided into three regions. The first is the isochronous volume, or the area within the PSF with the same arrival time, which is the bow-tie shaped region centered at the focus. Signals within this volume have had time to travel from the transducer to the target and back again. The second is the region above the isochronous volume which corresponds to times that precede the arrival of the target signal. The third is the region trailing the isochronous volume which corresponds to times that follow the arrival of the target signal. Phase aberration, and its effect on the transmitted and reflected wavefront, is illustrated in the second column of figure 1(B). Aberration, when caused by the skull, is generally depth-independent and target-brightness independent. Aberration induced by the skull conforms closely to a near-field aberration model, which is unlike the distributed aberration that occurs in structures like the body wall. Reverberation, shown in the third column of figure 1(B), can be categorized into two sources of image degradation. The first is multiply-reflected sound within the layered media that returns to the transducer, overlaid on top of sound returning from deeper ranges. The second is multiply-reflected sound that is transmitted beyond the layered media, contributing to a low-amplitude lengthening of the transmit pulse (Pinton *et al* 2011c). It has been shown that reverberation occurs in all three parts of the PSF due to different paths of multiple reflections (Pinton *et al* 2011c). This causes the hazing effect on top of the PSF, stemming from consecutive reflections caused by different impedance values between layers of the skull (Soulioti *et al* 2021). Since bone is a strong reflector, the distance from the skull to the transducer is also important, since reflections not only occur from within the skull region, but also between the skull surface and the transducer, causing reverberations in the area preceding the skull.

Techniques to quantify and correct for phase aberration have been established and validated in the context of brain therapy (Clement and Hynynen 2002, Almquist *et al* 2016, Leung *et al* 2021) and imaging (Jiang *et al* 2023, Kim *et al* 2023). In other studies (Tanter *et al* 1998, Ivancevich *et al* 2008, Pinton *et al* 2011b), the phase aberration was calculated by transmitting an outward propagating pulse from a point target at the focus to the transducer, and the corrected wave was re-transmitted from the transducer to the point target. It was reported that phase aberration correction increases contrast-to-speckle ratio by 13% and the detectability of cysts by 80% (Ivancevich *et al* 2006). In another study, phase correction improved the diagnostic ability of contrast enhanced transcranial imaging by detecting previously undetectable vessels (Ivancevich *et al* 2008). Since phase aberration correction depends on knowledge of the individual skull’s unique morphology, there has also been a focus on estimating the true sound speed in transcranial imaging using synthetic aperture imaging to mitigate the effects of refraction (Mozaffarzadeh *et al* 2021). Deep learning approaches have recently used neural networks to correct for the skull induced distortion from the acquired RF signal (Du *et al* 2020). In addition to phase and amplitude distortion, another factor affecting transcranial image quality is the presence of reverberation clutter. Clutter is a noise artifact that often obscures the regions of interest in ultrasound images, causing the hazing effect on top of the PSF. Reverberation clutter is caused by multiple reverberations, in this case by the impedance mismatch in the skull. Trailing clutter is caused by the impedance mismatch of the ballistic pulse. Clutter reducing methods, such as tissue harmonic imaging, have been applied to transcranial ultrasound to improve image quality in order to better visualize distinct brain regions (Tranquart *et al* 1999, Puls *et al* 2000). To simulate the effects of aberration on a PSF, clutter subtraction methods have been used to remove the effects of reverberation clutter, leaving the effects of aberration (Pinton *et al* 2011c). However, when using this technique, trailing clutter remains. Methods using a constant impedance can also be used to simulate isoimpedance; however, while this technique removes the trailing clutter, it does not remove all of the reverberation clutter, preventing the investigation of the relative contributions of aberration and reverberation on the PSF (Soulioti *et al* 2021). For the characterization of reverberation clutter in conventional ultrasound imaging, as well as the quantification of the contribution of aberration and reverberation to the degradation of the 3D PSF at both the fundamental and first harmonic frequency, phase aberration maps and PSFs have been previously used in human abdominal imaging (Pinton *et al* 2009, 2011c). However, the role of reverberations on transcranial image quality has not been investigated in detail.

Ultrasonic propagation through fine scale heterogeneities has been simulated previously with a finite difference time domain (FDTD) solution of the 2D and 3D linear wave equation (Mast *et al* 1997, Mast 2002). A numerical solution (Pinton *et al* 2009, Pinton 2021) to a full-wave equation has been developed that simulates the nonlinear propagation of waves in media with arbitrary or empirical frequency-dependent attenuation laws and variations in density and speed of sound. This full-wave nonlinear acoustic (Fullwave) code has been used to characterize reflections, aberrations, and standing waves caused by the skull to study therapeutic ultrasound for treatment of the brain (Pinton *et al* 2011b, 2011c, Soulioti *et al* 2019, Jones *et al* 2021, Aubry *et al* 2022). Bone has a complex micro-architecture consisting of an anisotropic heterogeneous porous structure with strong variations in its material properties (more than a factor of two variations in the speed of sound between bone and bone marrow). The average trabecular element is between 50 *µ*m and 150 *µ*m, with a separation that ranges between 0.5 mm and 2 mm, which is close to the typical wavelengths used in brain imaging. Even though conventional computed tomography cannot resolve the bone micro-architecture, it is used routinely to generate estimates of the overall acoustic properties in the skull bone. In other words, clinical CT imaging of the bone effectively acts as a homogenizing filter that blurs the bone micro-architecture. In this paper, we quantify aberration and reverberation clutter in 2D and 3D through the temporal acoustic window of the human skull using Fullwave simulations that are calibrated to experimental measurements. The acoustic properties of the skull sample are measured with computed tomography and converted into simulation maps that are calibrated to water tank experiments of the skull sample that are used to determine reverberation strength and root mean square aberration values. To elucidate the relative contribution of phase aberration and reverberation in transcranial image quality, two canonical imaging scenarios are explored: bright point targets at various imaging depths though the skull, and transcranial anechoic lesion imaging in calibrated tissue at different focusing depths. To make the anechoic lesion simulations more realistic, the average speckle brightness is calibrated in soft tissue using porcine liver. The combination of both analyses at multiple depths is used to characterize the dependency of these mechanisms (aberration, reverberation) on the nature of the target (reflectivity, presence of scattering environment) and the imaging depth.

## 2. Methods

**Figure 2.**
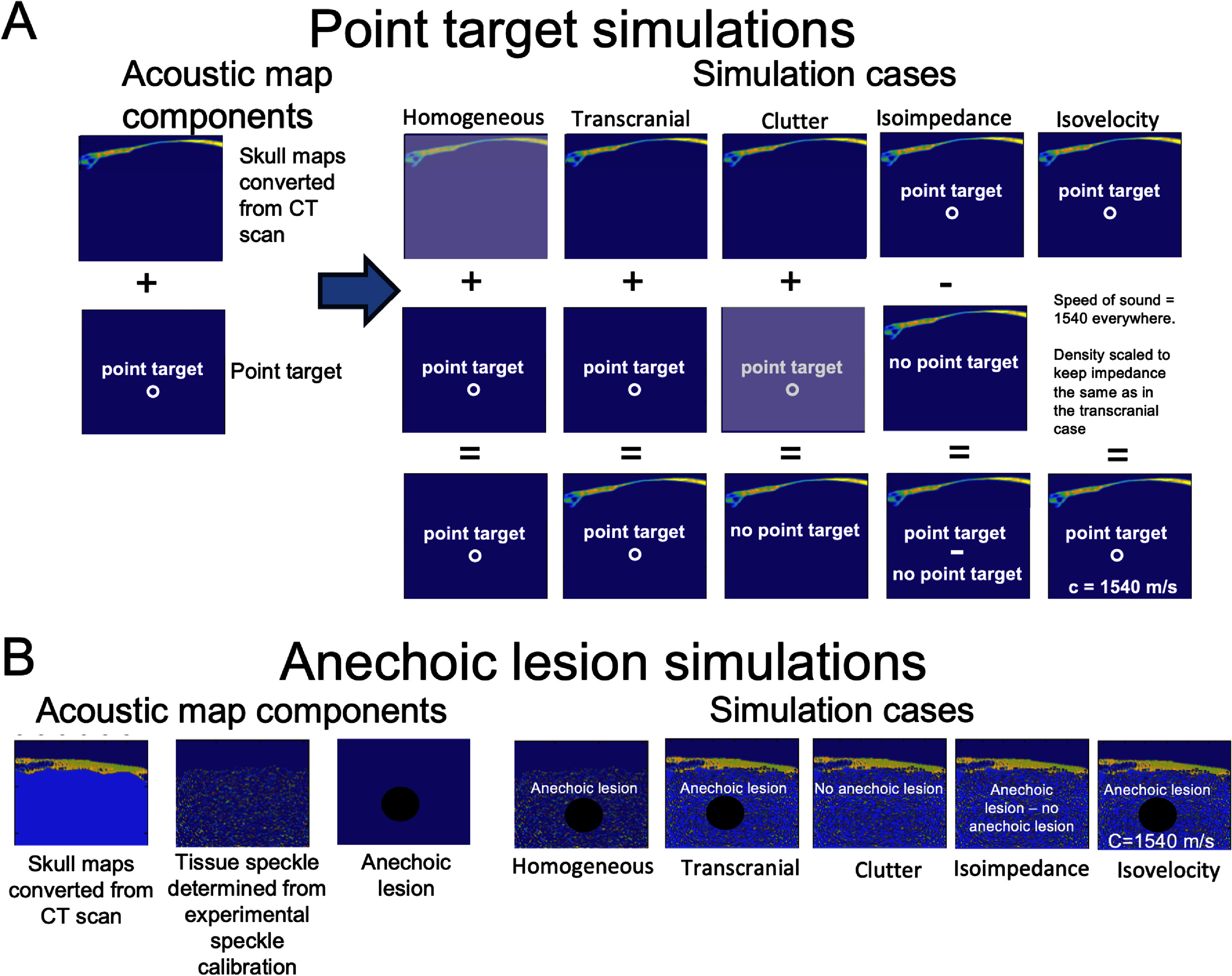
(A), Chart demonstrating the acoustic map components that were utilized for the point target simulations and which of those components were used to create the homogeneous, transcranial, and clutter simulations cases. The isoimpedance case is created by subtracting the clutter case from the transcranial case. The isovelocity case uses the same initial acoustic maps as the transcranial case; however, the speed of sound is set to 1540 m s^−1^ everywhere and the density map is then scaled to keep the impedance map the same. (B), Demonstrated the acoustic map components and the simulations cases for the anechoic lesion simulations.

The following section provides the detailed methodology of the performed *in silico* reverberation and aberration comparisons. More broadly, two main simulation target cases are performed using isovelocity and clutter subtraction methods, described in section 2.2.1. One is of a point target, in both 2D and 3D, described in sections 2.2.2 and 2.2.3, where the relative effects of aberration and reverberation on the PSF are compared. However, since target brightness can play a role in these effects, a second case is performed using an anechoic lesion, described in section 2.2.4. To create the anechoic lesion simulation, the lesion is surrounded by speckle. To allow for realistic speckle, a speckle calibration is performed experimentally. Both the point target and anechoic lesion simulations are performed transcranially using acoustic maps converted from CT scans and described in section 2.2.1. Since CT scans do not typically contain the skull microstructure, acoustic simulations using skull scans with and without the microstructure are compared, described in section 2.3. The accuracy of these skull maps is validated by comparing *in silico* and *in vitro* results in the same skull segment, described in section 2.4. A diagram representing how the different components were combined to create the isoimpedance and isovelocity maps is shown in figure 2. Important data pertaining to the article is available at the following DOI: 10.17615/7fbz-3d62 (Soulioti Danai *et al* 2025).

### 2.1. Simulation model

Simulations were performed using Fullwave2 and Fullwave2 3D (Pinton *et al* 2009, Pinton 2021), an ultrasound simulation tool that models wave propagation in an attenuating medium, using the quadratically nonlinear wave equation with multiple relaxations. This model is based on the following first-order pressure-velocity equations

\begin{equation*} \nabla_{1} p+\rho \frac{\partial \mathbf{v}}{\partial t} = 0\end{equation*}

\begin{equation*} \nabla_{2} \cdot \mathbf{v}+\kappa \frac{\partial p}{\partial t} = 0\end{equation*}

where *p* is the pressure, *ρ* is density, **v** is particle velocity, and *κ* is compressibility.

This simulation model accounts for nonlinearity through velocity-dependent spatial derivatives and for attenuation and dispersion by a convolution-based multiple relaxation model. It is implemented using a FDTD approach on a staggered-grid with perfectly matches layers on a parallel architecture, allowing for large, high-resolution simulations (Pinton *et al* 2009, Pinton 2021). Solutions are demonstrated in 2D and 3D for ultrasound imaging applications involving heterogeneous media simulating the human body (Pinton 2020), and compared to other ultrasound simulation tools for transcranial applications (Aubry *et al* 2022). In this work, for the 2D simulations described, a Linux Fedora 25 (v.4.10.13-200.fc25.x86 64) system running Intel Xeon® E5-2630 v4 processors at 2.20 GHz was used. For the 3D simulations, a Linux Ubuntu 20.10 system running Intel Core® i9-9820X processors at 3.30 GHz was used.

### 2.2. Acoustic propagation simulations

#### 2.2.1. Isovelocity and isoimpedance

To compare the effects of reverberation and aberration, isovelocity and isoimpedance simulations were performed. To do this, simulations with four different acoustic maps were performed. The first is a simulation of a target in a homogeneous medium, created using homogeneous density and velocity maps. The second and third are simulations with and without a target through the skull, where CT scans are used to create acoustic maps. To do this, the skull was segmented from the surrounding tissue or water using the Hounsfield units. Then, the remaining skull is scaled linearly, with the minimum speed of sound set to 1540 m s^−1^ and maximum set to 3000 m s^−1^. The same linear scaling is performed to create density maps, except with values between 1000 kg m^−3^ and 1850 kg m^−3^. These values are shown in table 1 and based on known acoustic values (Duck 1990). The attenuation maps are created using previously described methods where the porosity, which is the based on the inverse of density, was used (Aubry *et al* 2003). The transcranial case with the target shows the effects of the skull on the target compared to the homogeneous case while the transcranial case without the target produces a simulation of the clutter caused by the skull. The fourth simulation was a isovelocity case. To create the isovelocity case, the speed of sound was kept constant in the transcranial case (Pinton *et al* 2011c). Then, to maintain the same impedance, density was scaled so that impedance remained the same. To create the isoimpedance case, clutter subtraction techniques were used, where the clutter case, which is the transcranial case with no point target, is subtracted from the transcranial case with a point target (Pinton *et al* 2011c). This subtracts the reverberation clutter and removes the effects of multiple reverberations. Since multiple reverberation, in this case, is caused by impedance mismatch in the skull, this removes the reverberation clutter caused by the skull. Trailing clutter due to the point target is not subtracted out in the reverberation clutter images and therefore remains in our isoimpedance images.

**Table 1.** The acoustical properties of cortical bone in the skull used in the wave propagation simulations.

Material parameter	Cortical bone	Soft tissue
Density (*ρ*)	1850 kg m^−3^	1000 kg m^−3^
Speed of sound (*c*)	3000 m s^−1^	1540 m s^−1^
Nonlinear parameter (*B*/*A*)	7.6	7.6
Attenuation (dB MHz^−1^ cm^−1^)	2.8 or 13	0.3

#### 2.2.2. Point target simulations

2D transcranial simulations were performed to isolate the contribution of aberration and reverberation caused by the skull. To do this, simulations of a point target a 50 mm, emitting at 1.25 MHz to match the frequency of the 3D point target simulations described below, were performed. The homogeneous, transcranial, isoimpedance, and isovelocity cases were created as previously described and compared. Transcranial 2D simulations of a point target were performed for different focusing depths ranging from 30 to 70 mm. This configuration was used to test the hypothesis that for a shallow depth (∼30 mm), including regions within the cerebral cortex, the reverberation from the skull will contribute more to image degradation, whereas at larger depths that would include deeper structures like those within the temporal lobe, including the thalamus (∼65 mm), the aberration will play the main role in image degradation. Additionally, simulations to analyze the effects of harmonic imaging transcranially were performed using a point target at 50 mm in depth. Both the fundamental and first harmonic were compared. All B-mode images are displayed in dB and scaled to their local maximum.

#### 2.2.3. 3D simulations

The effects of the skull in ultrasound imaging on a point target were also explored in the 3D simulation space. A custom designed 1024-channel 2D sparse array emitting at 1.25 MHz was used (McCall *et al* 2023). This array was designed for human transcranial imaging and has a 65 mm circular aperture, with elements arranged in a sparse tapered spiral. This allows for the transmit of an apodized beam with high focal gains and low side lobes (Diarra *et al* 2012).

A 3D CT data set was obtained from a healthy volunteer made with a Siemens CT VA1 (Siemens Medical Solutions, Erlangen, Germany) machine with a slice thickness of 1 mm, and an in plane resolution of 0.78 mm. The scan was performed at 120 kVp and using a B45 reconstruction kernel. Using a temporal window, a 3D segment of the skull was selected for imaging and converted from its original Hounsfield units to density, speed of sound and attenuation maps. The maximum and minimum values for the above properties of the skull were selected in the same way as for the 2D simulations. Run times for the 2D sims were 1 h per simulation at 20 points per wavelength compared to approximately 8 h per simulation at 12 points per wavelength for the 3D simulations.

#### 2.2.4. Anechoic lesion in a scattering medium

To model a realistic brain imaging scenario where scattering tissue is present and imaging targets are not as bright, the same isovelocity and isoimpendance simulations were performed on an anechoic lesion of a 1 cm diameter. To accurately mimic tissue, this lesion was inserted in an experimentally calibrated scattering medium at different imaging depths of 3, 4, 5, 6, and 7 cm. To match the experimentally calibrated tissue brightness, 2.5 MHz was used to match the frequency of the Phillips ATL P4–1 probe (Philips Healthcare, Bothell, WA, USA) that was used for experiments. Tissue was simulated by inserting multiple scatterers with a density of 15 scatterers per wavelength cell, with a density variation in the order of 0.5% compared to a background density of 1000 kg m^−3^. A single pixel scatterer has a size of 51 *µ*m. Using this speckle distribution, anechoic lesions, where scatterers are completely absent, can be inserted in our acoustical property input maps. Lesions were also set to be non-attenuating, while the scattering medium has an attenuation value equal to 0.5 dB cm^−1^ MHz^−1^, which is typical for soft tissue (Maklad *et al* 1984). To successfully resolve a circular anechoic lesion with a radius of 5 mm transcranially, a multi-focused emission approach is used, where at 2.5 MHz, 48 individual foci are used, spaced 0.4 mm apart laterally, at a constant focal depth equal to the center of the lesion. The raw data acquired is beamformed and then combined into a single B-mode, with each lateral area corresponding to its respective focus. Isoimpedance and isovelocity versions of these simulations were performed to evaluate how aberration and reverberation affect the ability to resolve the lesion and its contrast-to-noise ratio (CNR), calculated as the mean inside the lesion minus the mean of the background, divided by the standard deviation of the background.

To determine the relative magnitude of backscattered signals, or speckle brightness, to the reverberation from the skull, calibration was performed using soft tissue. The pressure output as a function of voltage for the transducer used in experiments (P4–1 at 2.5 MHz) was measured with a HNC-0200 needle hydrophone (Onda Corp.). For a transmit of 5 V, a pressure of 259 kPa is emitted along the midline of the transducer. Data from fresh, degassed porcine liver immersed in water were also acquired, using a focused wave with a fixed focus at 50 mm. From the beamformed A-line, we measured the average brightness of the liver tissue, while averaging through 10 different elevation slices and 100 frames per elevation position. This yielded a reference brightness of −33 dB at the focus.

### 2.3. Effects of skull homogenization

For the simulations used to characterize the effects of reverberation and aberration, CT scans are used to create acoustic maps of the skull. Since CT scans often do not contain the microstructure of the skull, the difference between simulation results using acoustic maps created from scans with and without the microstructure were compared. Previous studies have shown that skull homogenization has large impacts on ultrasound transmission (Robertson *et al* 2018). Thus, prior to quantifying the main degradation mechanisms transcranially, the effects of this homogenization on reverberation were investigated.

**Figure 3.**
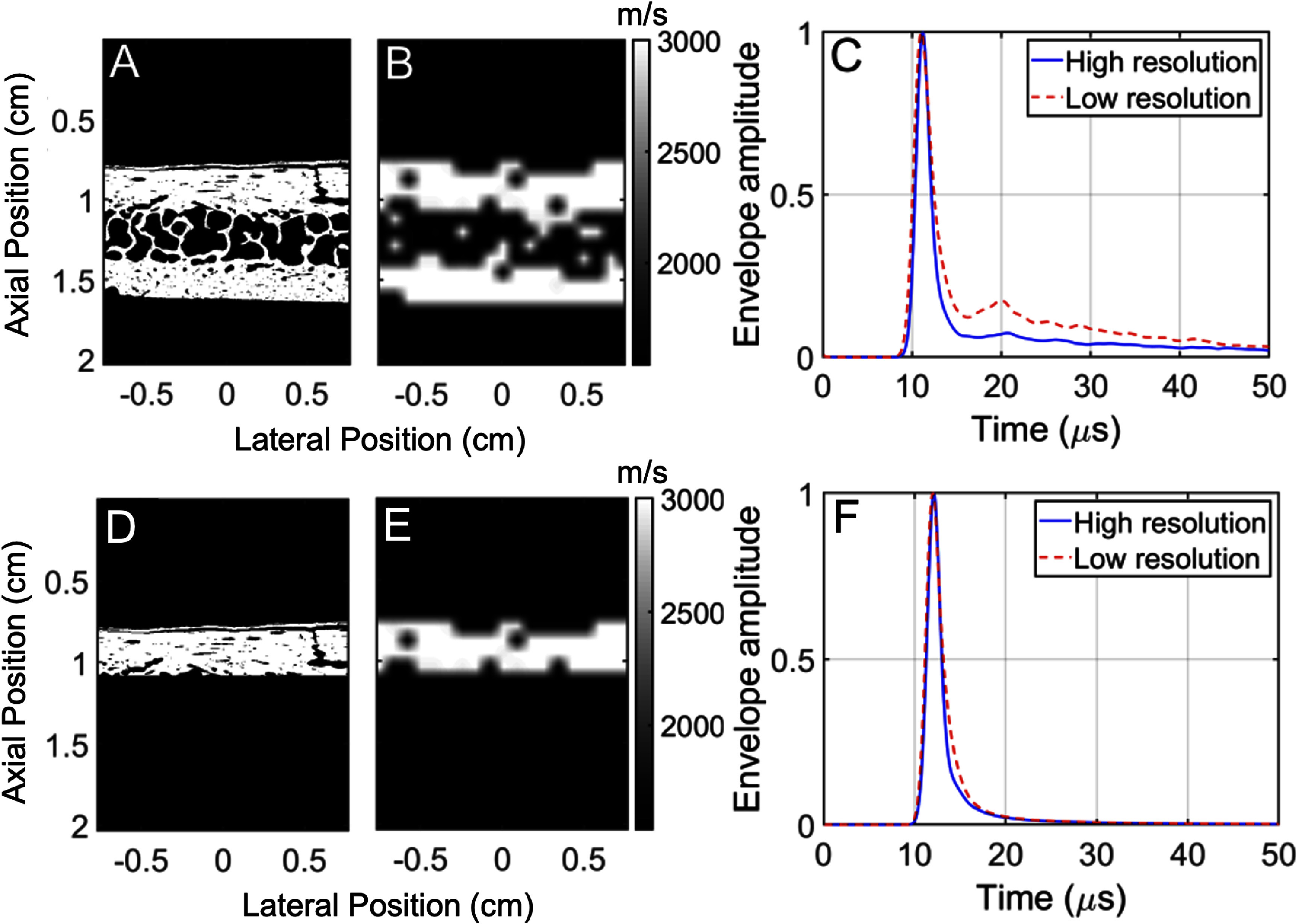
(A), A 2D section of the 3D synchrotron images with an isotropic 10 *µ*m resolution. (B), The same data set after it has been downsampled to a resolution of 800 *µ*m and then interpolated back to a 10 *µ*m resolution. (C), The envelope detected amplitude of the imaging pulse after propagating through the high resolution map (solid) and the low resolution maps (dashed) shown in (A) and (B). (D), A 2D section of the thin 3D synchrotron data with an isotropic 10 *µ*m resolution. (E), The same data set after it has been downsampled to a resolution of 800 *µ*m and then interpolated back to a 10 *µ*m resolution. (F), The envelope detected amplitude of the imaging pulse after propagating through the high resolution map (solid) and the low resolution maps (dashed) shown in (D) and (E).

Previously measured bone micro-architecture of a human skull imaged with 3D microtomography at a 10 *µ*m resolution (Pinton *et al* 2011a) was used to determine whether brain scans with the resolution of a conventional CT is sufficiently accurate to model the reverberating properties of the cortical and diploë regions of the skull bone. To obtain a high resolution scan of a skull that contains the best representation of the microstructure to act as a ground truth for comparison with scans without the microstructure, a synchrotron was used, where electrons were accelerated in a linear accelerator (LINAC) followed by a booster synchrotron, reaching energy levels of 6 GeV. These electrons then went into a storage ring (844 m circumference) with a current of 40 mA. Figure 3(A) shows a 2D section of the synchrotron data with an isotropic 10 *µ*m resolution. Figure 3(B) shows the same data set after it has been downsampled to a resolution of 800 *µ*m, which is the typical resolution of CT scanner, and then interpolated back to the original 10 *µ*m resolution so that the microstructure is homogenized. This data was then converted to maps of the acoustical properties of bone, as shown in table 1. In this section the attenuation of the cortical bone was set to 2.8 dB MHz^−1^ cm^−1^. In the following sections, where low resolution images are used, the attenuation is set to 13 dB MHz^−1^ cm^−1^ to compensate for the lack of microstructure, as previously calculated and validated using hydrophone measurements on the same skull sample used for the synchrotron scan (Pinton *et al* 2011a).

Simulations were performed at 1 MHz, a frequency with overlap between human transcranial therapy and imaging applications with a wavelength within the trabecular element separation range, to match previously performed experimental attenuation calibrations on the skull piece used in the synchrotron scan (Pinton *et al* 2011a). A 1 MHz 2 cycle Gaussian enveloped pulse was transmitted into this medium from a planar surface at 0.4 cm depth. The simulated signal was recorded following propagation through the bone, along the plane at depth 1.65 cm for a duration of 50 *µ*s. This received signal was envelope detected and averaged over the plane. It is plotted in figure 3(C) as a solid line for the high resolution medium and as a dashed line for the low resolution medium. The ballistic pulse can be observed at approximately 12 *µ*s and the reverberating coda can be observed following the pulse. The low-resolution map results in a significantly more reverberating medium, with a coda amplitude that is almost twice as large as observed in the high-resolution map. This indicates that when propagating through thick cortical-diploe-cortical regions of the skull, the microstructure contributes significantly to transcranial reverberation.

However, the bone sample in figures 3(A) and (B) comes from a thick part of the skull, with a thickness of approximately 8–9 mm. The temporal window that is typically used for imaging, on the other hand, is much thinner, between 1 and 3 mm, and it is composed almost entirely of cortical bone. For the sake of consistency, the same map was truncated at a depth of 1.1 mm so that the only cortical bone with a thickness of 3 mm is included (shown in figures 3(D) and (E)). The envelope detected pulse for this more realistic map as measured at 1.65 cm is shown in figure 3(F). In this case there is an excellent agreement between the reverberation resulting from the high-resolution and low-resolution maps. This indicates that in dense cortical regions, the small amount of microstructure does not contribute significantly to transcranial reverberation. Consequently, clinical resolution images should be sufficient to determine the reverberation properties in regions with less microstructure, like the temporal region of the skull.

### 2.4. Skull map calibration

To validate the accuracy of the skull maps used, *in silico* and *in vitro* transcranial results were compared using the same piece of skull. Experiments using a P4–1 probe at 2.5 MHz were performed through an adult male human skull specimen in a water tank with a 250 *µ*m nylon wire (Berkley Nanofil) as an imaging target. The temporal region was used, since it usually provides a representative acoustical window for transcranial imaging. The skull was degassed in a vacuum chamber for 24 h prior to the experiment to remove any residual air that might compromise image quality. A 3D printed custom holder that holds the P4–1 transducer and the skull piece in a registered manner was used for the experiment. The wire target was placed at 50 mm of depth from the transducer, and a fixed focus pulse was emitted, focusing at the depth of the target. The wire target was placed perpendicular to the long axis of the transducer in order to act as a point target in the imaging plane. The PSF acquired for the target was then compared with a PSF of the same target in water to calculate the root mean square difference value between the backscattered profiles of the wire transcranially and in the homogeneous medium.

**Figure 4.**
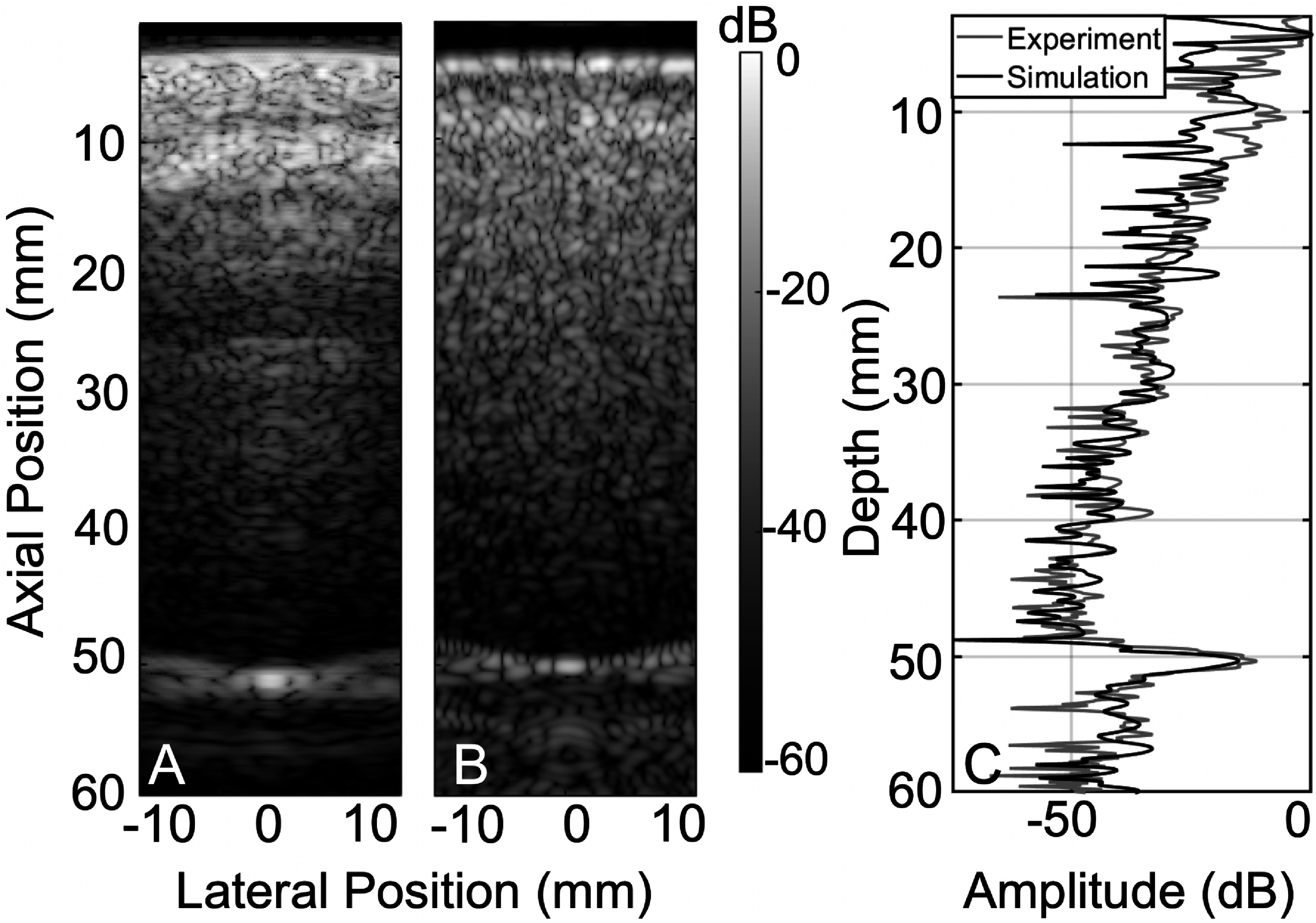
(A), Experimental B-mode for a 2.5 MHz emission through a human skull. (B), Simulation B-mode using a skull map converted from a CT scan of the same skull specimen. (C), Reverberation curves for images (A) and (B).

To maintain registration and calibration, the same transducer holder with the skull piece attached was scanned using the CT component of a Super Argus 4R PET/CT (Sedecal, Inc, Madrid, Spain) with an in-plane and slice resolution of 0.105 mm. Imaging was performed on the higher end of standard voltage parameters, which range between 40 and 120 kVp, and reconstructed using the Feldkamp algorithm. References of air and water were also scanned simultaneously to linearly convert the CT maps in Hounsfield units to density maps, assuming values of 1.225 and 997 kg m^−3^ for the two respectively, and yielding a conversion formula of *dmap* = (*HUmap* + 15.9)/13. The 2D slice corresponding to the middle of the physical transducer was chosen for the simulations. To ensure calibration, reverberation curves between simulation and experiment were compared. As shown in figure 4, the reverberation pattern is acquired by laterally averaging the beamformed images of both the simulation and experimental counterparts and then plotting them as a function of depth. The aberration values were measured by calculating the root-mean-square (RMS) time shift of the backscattered profiles of the target both transcranially and in a homogeneous medium (water). These values were 53 ns RMS for the experiment and 57 ns RMS for the simulation. Previous studies have measured this aberration RMS value to be up to 47.5 ns RMS in the intercostal spaces (Hinkelman *et al* 1997), ranging between 25.6 to 75.9 ns RMS in the abdominal wall (Hinkelman *et al* 1994, 1998), and up to 53 ns RMS *in vivo* (Trahey *et al* 1991, Freiburger *et al* 1992) and 66 ns RMS in autopsy specimens in human breast tissue (Hinkelman *et al* 1995). Compared to these values, this specific skull specimen is mildly aberrating.

**Figure 5.**
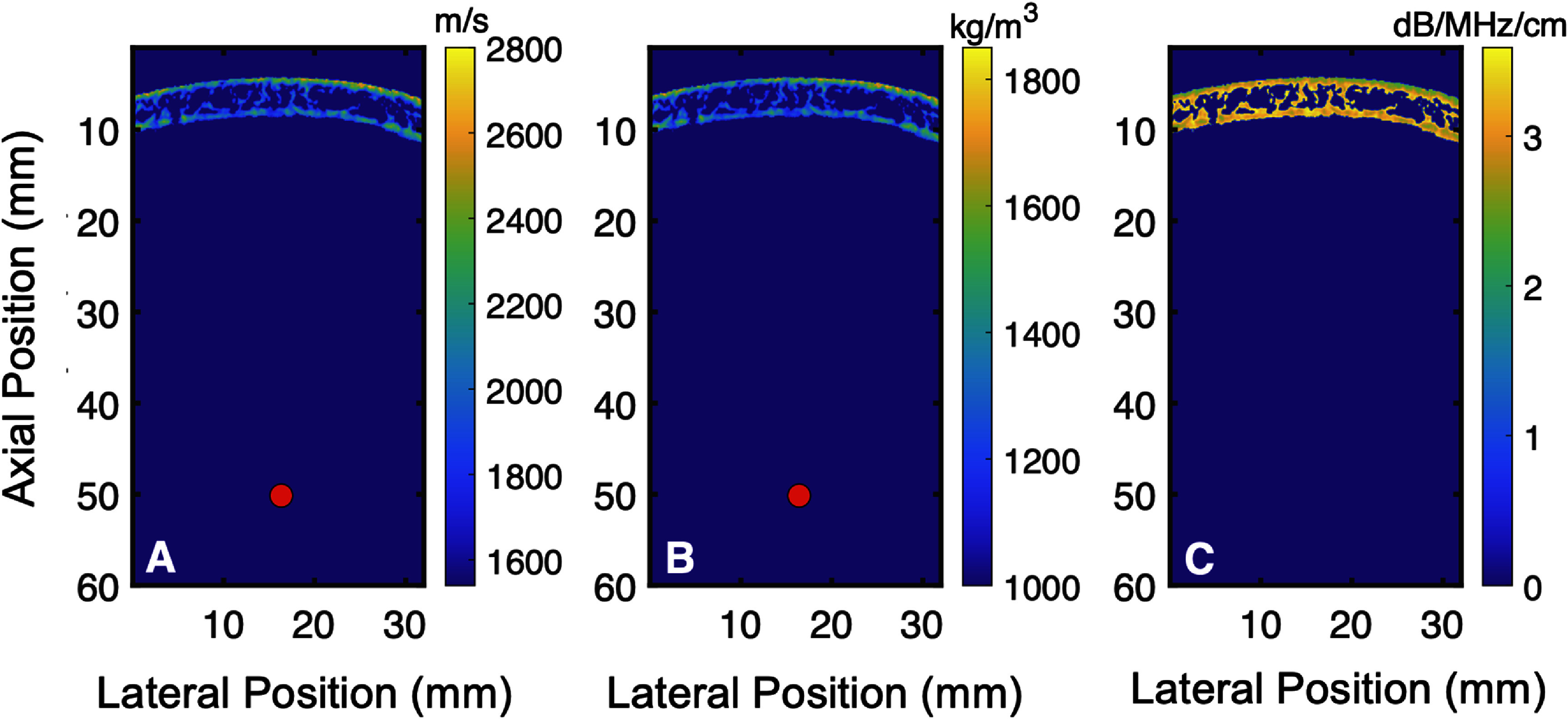
Input acoustical field maps for Fullwave2: sound speed (A), density (B) and attenuation (C). The size of the point target at 50 mm is exaggerated.

Simulations were performed at a frequency of 2.5 MHz to allow for comparison with experimental results, with an initial pulse pressure of 0.3 MPa. Two realizations were explored, one in a homogeneous medium (uniform sound speed of 1540 ms^−1^ and density of 1000 kg m^−3^) and one transcranially, using the same acoustical maps shown in figure 5. Attenuation was set to zero for the homogeneous medium and was scaled inversely to density for the skull as previously described (Aubry *et al* 2003) and shown in figure 5(C). Non-linearity values were not used for the calibration here, so were kept at zero; however, the nonlinear components for transcranial propagation were previously validated experimentally (Pinton *et al* 2011a). It has also been shown that clutter subtraction works both with and without nonlinearity components set to zero (Pinton *et al* 2009).

## 3. Results

### 3.1. Isolation of the components of image degradation

**Figure 6.**
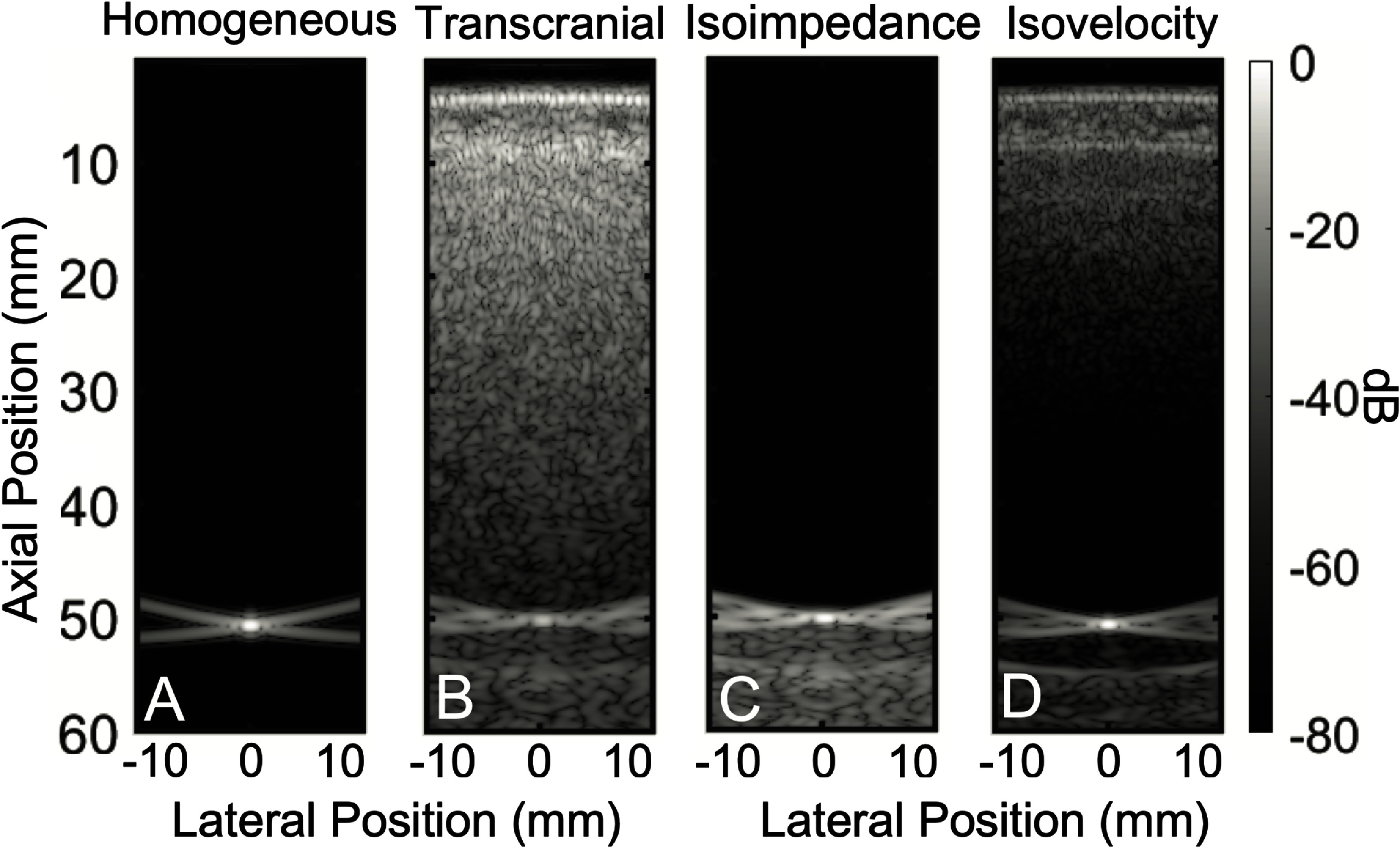
(A), Homogeneous B-mode of a target at 50 mm. (B), Transcranial B-mode of the target. (C), Isoimpedance B-mode where the clutter has been subtracted and multiple reverberations are removed. (D), Isovelocity B-mode where all effects of aberration are removed.

To quantify the contribution of aberration and reverberation in the total clutter produced by the insertion of the human skull, using the acoustic skull maps shown in figure  5, isoimpedance and isovelocity B-mode images were generated *in silico*. The homogeneous case is shown in figure 6(A). Isoimpedance B-modes result in the elimination of the effects of reverberation, whilst preserving aberration. This process can be divided into two steps, propagating a wave through the acoustical maps alone, without the presence of a scatterer, and then linearly subtracting it from the heterogeneous B-mode image shown in figure 6(B) to obtain the isoimpedance B-mode shown in figure 6(C). To generate the isovelocity B-mode, where aberration is removed and reverberations persist, sound speed is set to be constant at a value of 1540 m s^−1^ through the entire field, while density is scaled appropriately so that impedance retains its original value, yielding image figure 6(D).

### 3.2. Depth dependency of reverberation and aberration

**Figure 7.**
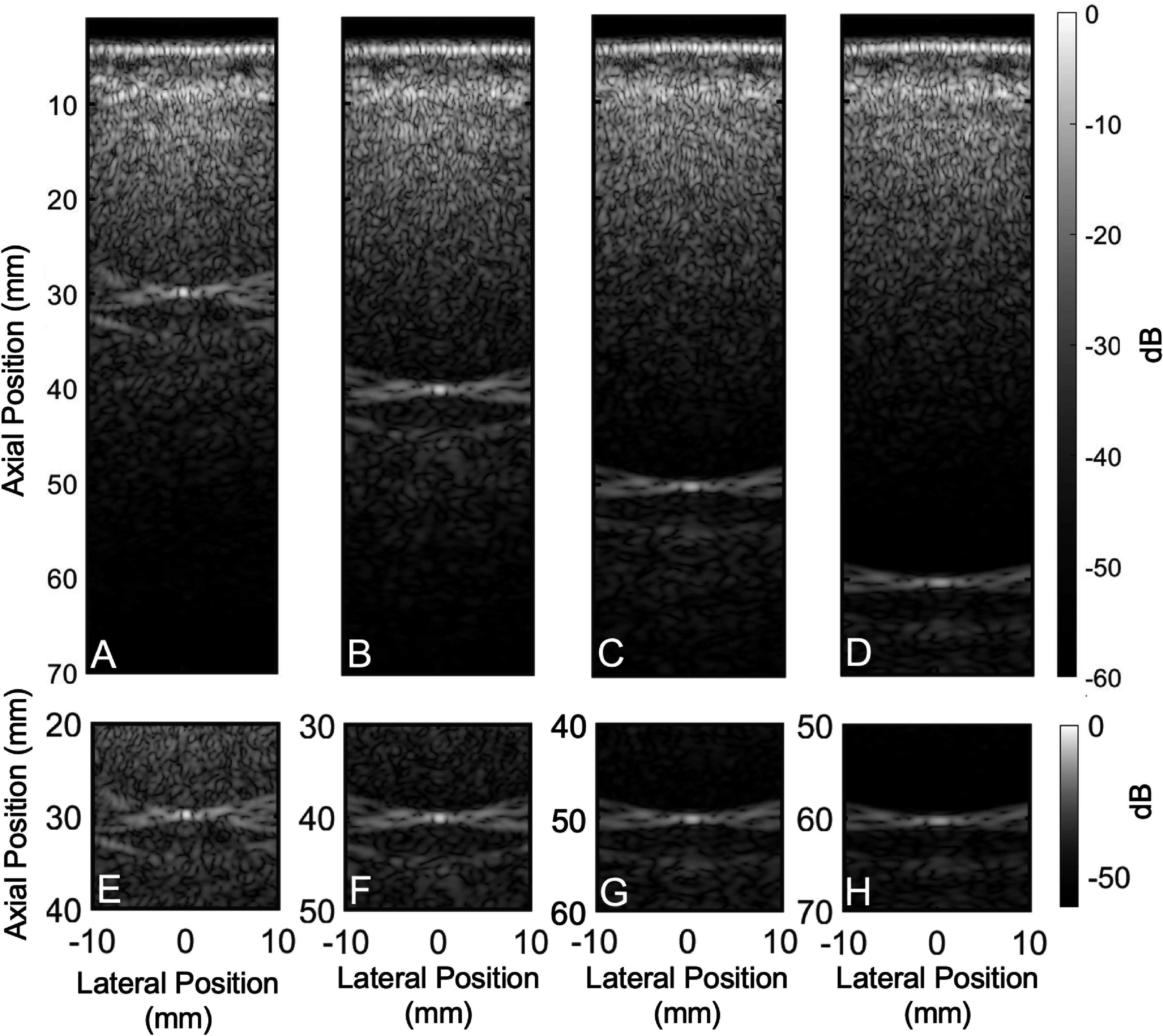
A point target simulated at (A) 30 mm, (B) 40 mm and (C) 50 mm and (D) 60 mm, transcranially, with zoomed in images of the points targets to allow for direct comparison across depths shown in (E) for 30 mm, (f) for 40 mm, (G) for 50 mm, and (H) for 60 mm. Units are in normalized dB.

To test whether the contribution of reverberation changed with depth, simulations with point targets at different depths were performed. This is indeed corroborated in figure 7, where we see that in case (A), reverberation severely impacts the quality of the PSF at 30 mm. The preceding region should be black; however, due to the presence of reverberation, there is significant clutter present in the regions below the skull, severely impacting the quality of the PSF at shallower depths. In image (B), the effect of the clutter on the PSF at 40 mm decreases but is still evident. Finally, in image D, at a depth of 60 mm, reverberation has subsided to values below −60 dB, due to attenuation of the signal. Note, however, that trailing clutter, which appears following the PSF, persists at large depths.

Table 2 summarizes the average dB values of the preceding regions, isochronous volume and trailing regions of the PSFs for different depths of the scatterer, as also shown in figure 7. Aberration values were measured for different depths through the isoimpedance RF data, where the effects of reverberation are removed, and no significant variation was found with depth, for example RMS aberration was measured at 77, 75 and 71 ns for 30, 50 and 70 mm of depth, respectively.

**Table 2.** Mean magnitude of three PSF regions in dB for different scatterer depths.

	3 cm	4 cm	5 cm	6 cm
Preceding region	−33	−42	−52	−60
Trailing region	−34	−38	−42	−45
Isochronous volume	−34	−35	−38	−39

**Figure 8.**
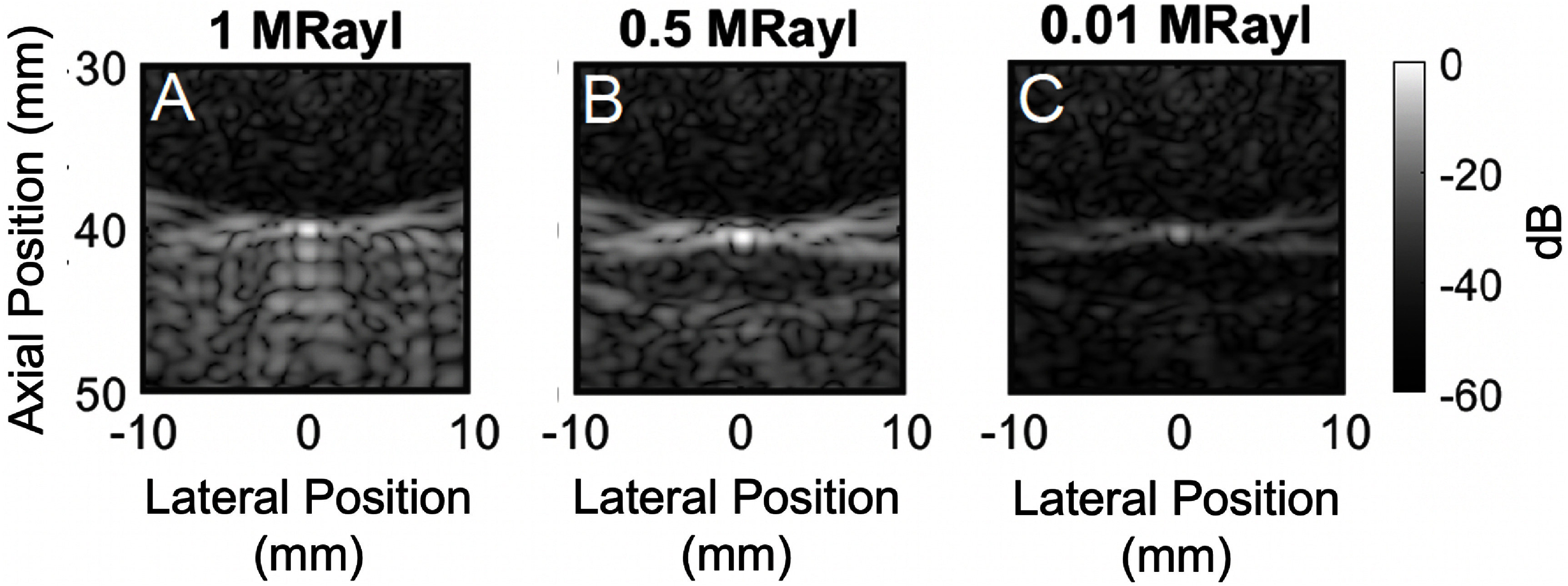
Point targets that have their sound speed scaled by (A), 10%, (B), 20% and (C) 50% with respect to their original value, displayed in dB.

In figure 8, the same simulation shown in figure 7(B) for a target at 40 mm is performed, but the target impedance is changed with respect to the background to values of 1, 0.5 and 0.01 MRayl. There is no difference in either aberration values or multiple reverberations originating from the skull itself, however trailing clutter scales proportionally with increasing impedance mismatch. For a low impedance mismatch, trailing clutter is at the same amplitude level as the preceding region, but as impedance increases, so does the trailing clutter, reaching values only 5 dB lower than the PSF maximum value for an impedance mismatch of 1 MRayl.

### 3.3. Coherence degradation in transcranial imaging

Coherence curves as well as Lag-One Coherence measurements are performed for the different anechoic lesion simulation cases shown in figure 13. Spatial coherence is sensitive to wavefront degradation due to aberration as shown in literature (Lediju *et al* 2011). It has also been shown that in the presence of reverberation clutter, rapid decorrelation in spatial coherence at very short lags occurs (Pinton *et al* 2014).

**Figure 9.**
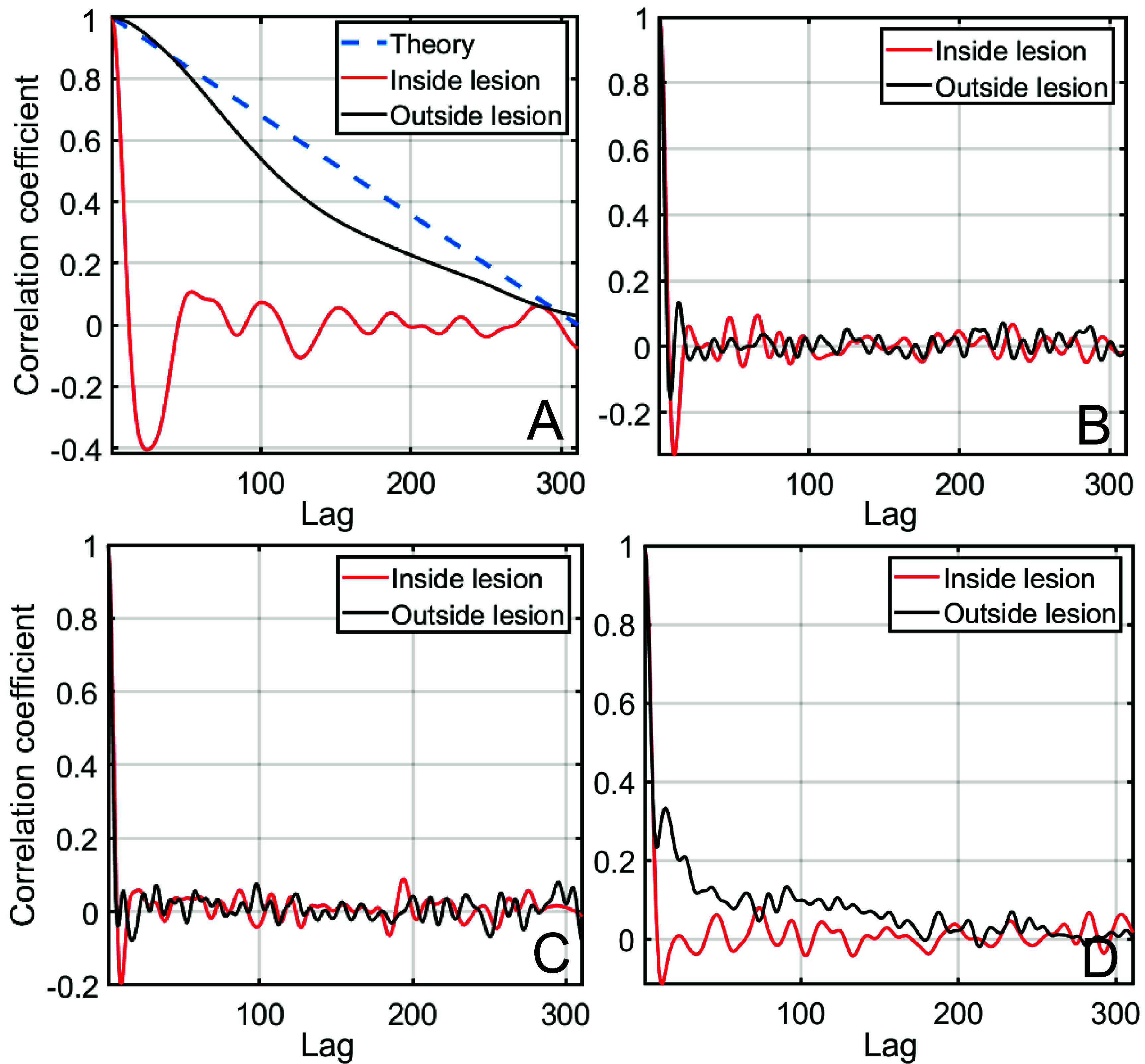
(A), Coherence curves for a homogeneous emission inside and outside the anechoic lesion. Theoretical curve for the ideal case is also shown as reference. (B), Coherence curves for a transcranial emission, (C) with the effects of phase aberration removed (isovelocity) and (D) with the effects of reverberation removed inside and outside the anechoic lesions. All plots generated from lesions at a depth of 3 cm.

In figure 9(A), the spatial coherence curves are shown for the homogeneous B-mode at a shallow depth of 3 cm. The correlation coefficients are calculated as a function of inter-element lag for RF data corresponding to the anechoic interior of the lesion, the surrounding tissue outside of the lesion, and lastly the ideal theoretical case as described by the Van Cittert–Zernike theorem for a random scatterer distribution (Mallert and Fink 1990). For tissue modeled as a uniform distribution of sub-resolution scatterers, the coherence curve matches theoretical predictions from the Zernike curve. On the other hand, for locations that are inside the anechoic lesion, there is a rapid decorrelation. This is consistent with the interpretation that since the anechoic portion of the image does not reflect sound, any sound that is beamformed to that location must be decorrelated since it was reflected by off-target sources. A more comprehensive description of this behavior can be found in the (Pinton *et al* 2014). The coherence curve inside the lesion should, therefore, always be decorrelated so this curve acts as a control for the other three cases (figure 9).

In figure 9(B), the coherence curves for the transcranial case are shown. (C) and (D) Show the isovelocity and isoimpedance cases, respectively. All curves are derived from lesions at 3 cm of depth. Figure 9(B) shows that the skull significantly degrades the coherence estimates from tissue. In fact, the coherence of tissue is just as poor as the coherence of the anechoic lesion, i.e. both are almost completely incoherent. Removing aberration (figure 9(C)) does not improve the tissue coherence. However, the removal of reverberation (figure 9(D)) significantly improves the coherence of the tissue signal and the short-lag coherence values reach 0.35. In other words, at this depth, tissue coherence is less affected by removing aberration than removing reverberation.

### 3.4. Effects of harmonic imaging

**Figure 10.**
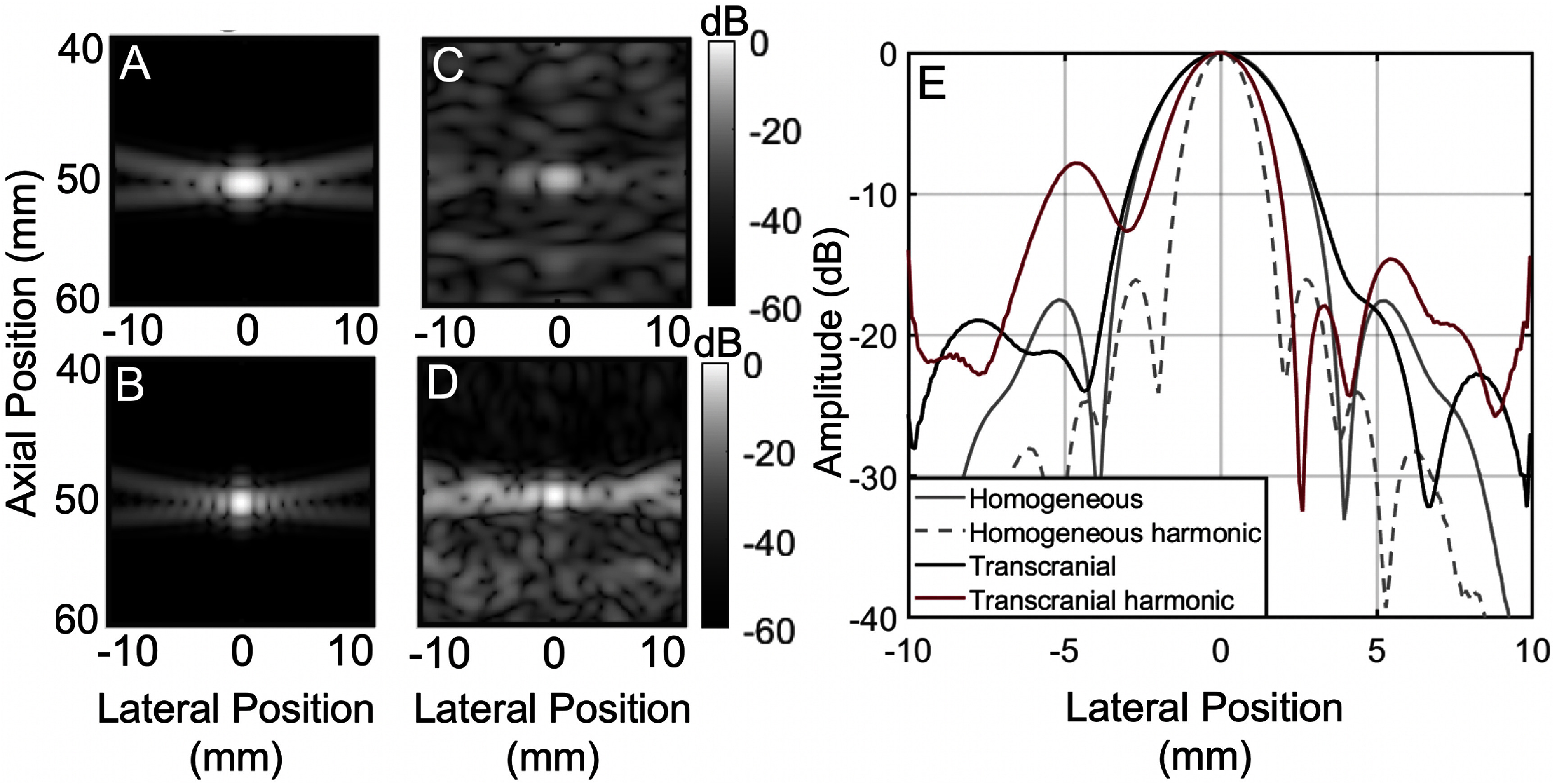
PSFs from an unapodized transducer in a homogeneous medium (left) and transcranially (right) for a target at a depth of 50 mm, for the fundamental frequency of 1.25 MHz (A), (C) and the first harmonic frequency of 2.5 MHz (B), (D). The PSFs have been normalized relative to their peak. All scales have units of decibels. (E), Beamplots for PSFs (A)–(D).

The effect of harmonic imaging on the PSF was also explored *in silico*. The RF data was filtered at the fundamental and first harmonic, as shown in figures 10(A) and (B) respectively for a homogeneous reference case and figures 10(C) and (D) for the transcranial case. Figure 10(E) shows the beamplots for all cases (A)–(D). Harmonic imaging reduces the width of the main lobe in both homogeneous and transcranial cases. As far as reverberation clutter is concerned, harmonic imaging decreases its amplitude by an average of 15 dB.

### 3.5. 3D transcranial simulations

**Figure 11.**
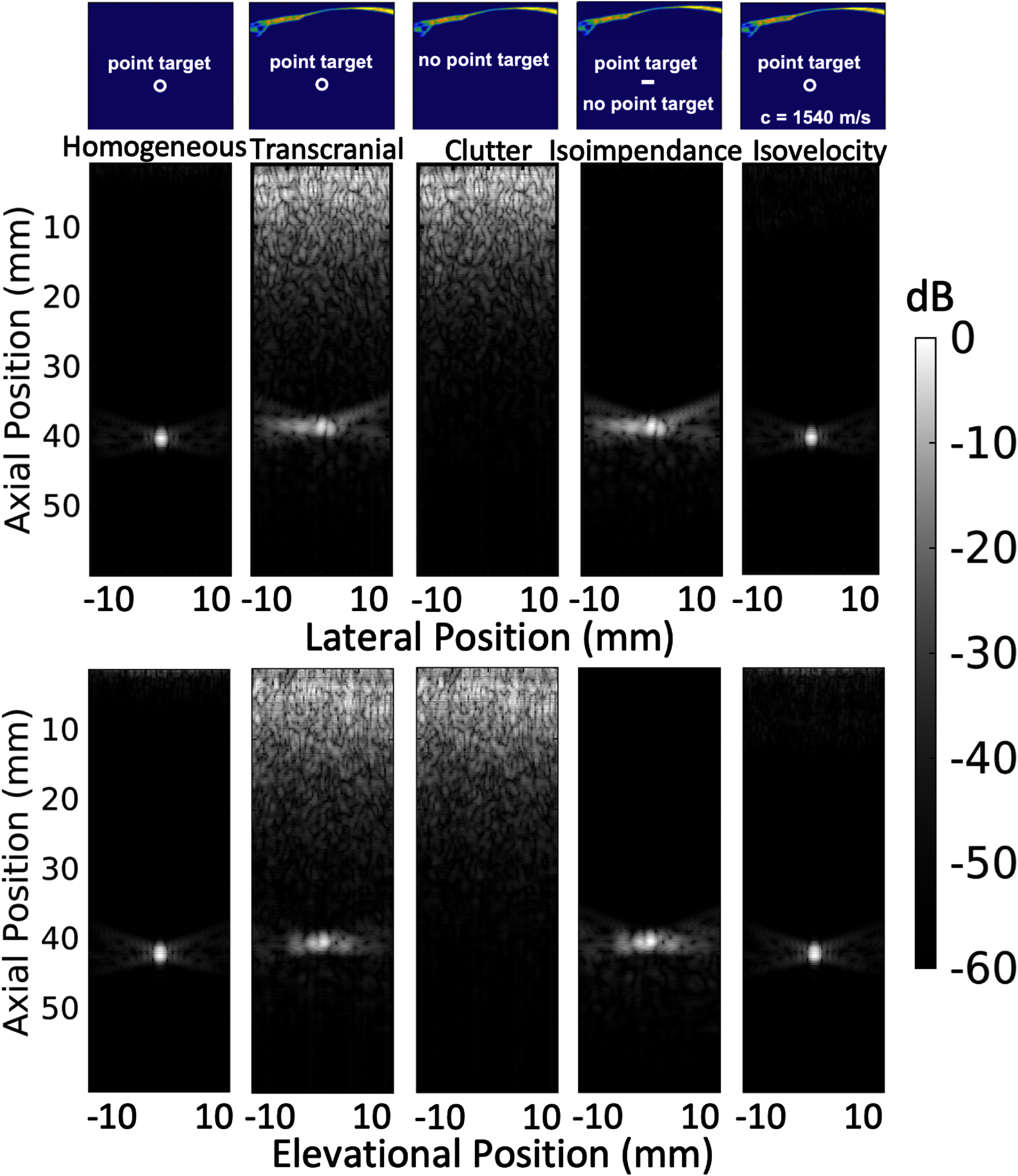
B-mode images for 3D simulations for the central lateral (second row) and elevational (third row) slices, corresponding to different imaging scenarios shown schematically (first row).

Similarly to the 2D case, as the wave propagates, part of the energy is trapped in multiple reverberations between the bone surfaces and the transducer. In 3D, however, the extra dimension can potentially modify the reverberation and aberration effect. Part of the pulse is reflected by the point scatterer at the focus. The signal is then measured at the transducer surface and used for 2D delay and sum beamforming operations. 3D simulations were performed at 8 cm deep, 6.5 cm wide, and 6.5 cm in elevation, which corresponds to the physical dimensions of the sparse matrix array (McCall *et al* 2023). The transducer emits a focused imaging pulse towards a bright spherical point target at a depth of 40 mm. The PSF can be determined by making an image of the point target where the total PSF includes both the effects of phase aberration, reverberating clutter, and trailing clutter in three dimensions. The PSFs for the different cases are shown in figure 11. The PSF for a spherical target in a homogeneous medium is shown in the leftmost column of figure 11. The PSF with the skull bone in place with both the effects of aberration and reverberations included is shown in the second column. The clutter in the absence of a target is shown in the middle column. Since there is no point target, this PSF is calculated entirely from signal that is reverberating between the bone and the transducer. The fourth column shows the result of the subtraction of column three from column two, i.e. the subtraction of clutter from the PSF. Note that in the area preceding the isochronous volume there is no signal. However there is signal following the PSF. This corresponds to the trailing distortions of the ballistic pulse. Removing the effects of aberration by setting the speed of sound to be constant and scaling density appropriately to preserve impedance, yields the image in the fifth column of figure 11. Qualitatively, removing phase aberration restores the shape of the PSF completely. Each B-mode is scaled to the maximum of the PSF without the skull.

To further quantify the contribution of each degradation mechanism, measurements of the average amplitude in absolute dB units of different PSF regions is summarized in table 3. Interpreting table 3, and using the homogeneous values as a reference, inserting the skull in the heterogeneous case increases the dB values of the preceding region by 40 dB. Removing the effects of phase aberration restores the amplitude of the preceding and trailing regions by 28 and 2 dB respectively, and the isochronous volume by 9 dB. Removing the effects of reverberations restores the amplitude of the preceding region by 85 dB and the trailing region by 4 dB. For the isoimpedance case, the mean values of the preceding region is lower even than the homogeneous case.

**Table 3.** Average magnitudes in absolute dB values of the three regions in different cases for a spherical target at 40 mm.

	Preceding	Trailing	Isochronous
Homogeneous	55	93	120
Heterogeneous	95	93	108
Isoimpedance	10	89	108
Isovelocity	67	91	117

### 3.6. Effects of target brightness on aberration and reverberation

**Figure 12.**
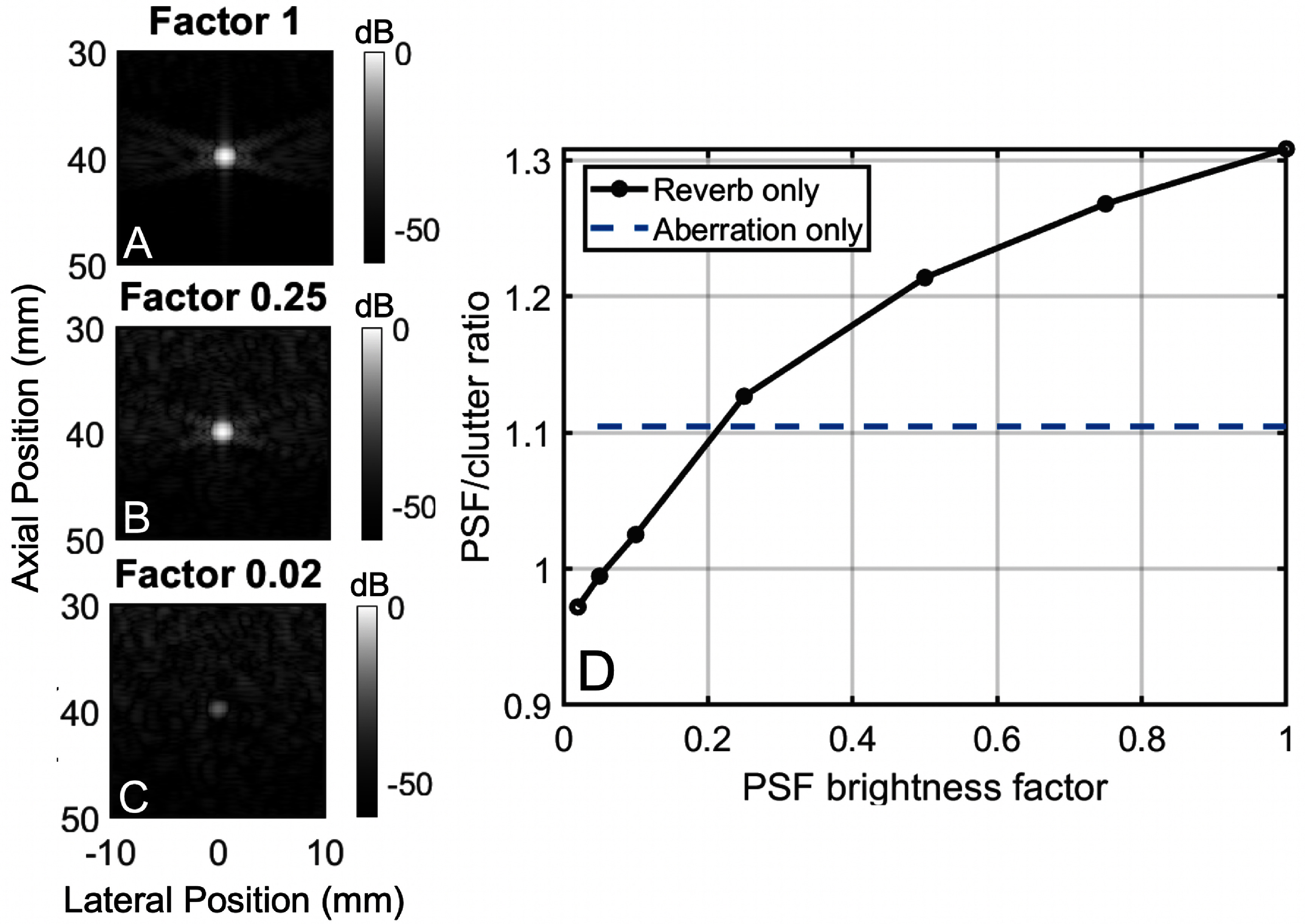
(A)–(C), Zoomed-in isovelocity B-mode images of targets at 4 cm depth that have their brightness scaled by a factor of 1, 0.25 and 0.02. (D), Ratio of the mean amplitude of the isovelocity PSF over the mean amplitude of the background clutter as a function of target brightness.

To compare the effects of aberration and reverberation on point targets of different brightnesses, three 3D simulation were compared for three different point target brightnesses, scaled by a factor of 1 (figure 12(A)), 0.25 (figure 12(B)), and 0.02 (figure 12(C)). The PSF to clutter ratio was calculated for each case as the ratio of the PSF mean amplitude, after removing the effects of phase aberration, over the mean amplitude of the background clutter. Across the three cases, the PSF to clutter ratio when only aberration is present remains constant; however, when only aberration is present, the PSF to clutter ratio increases with PSF brightness (figure 12(D)). Therefore, aberration is not affected by target brightness while reverberation is. At lower target brightnesses, the PSF to clutter ratio is much lower during the reverberation only case, meaning the image is worse. As target brightness increases, so does the PSF to clutter ratio, meaning that reverberation has greater effects at lower brightness amplitudes. Aberration only degrades the isochronous volume, whereas reverberation degrades a larger area of the PSF. Therefore, small degradations in amplitude can have a larger effect on overall image quality due to a larger convolutional area.

### 3.7. The relative importance of clutter and aberration in image quality

**Figure 13.**
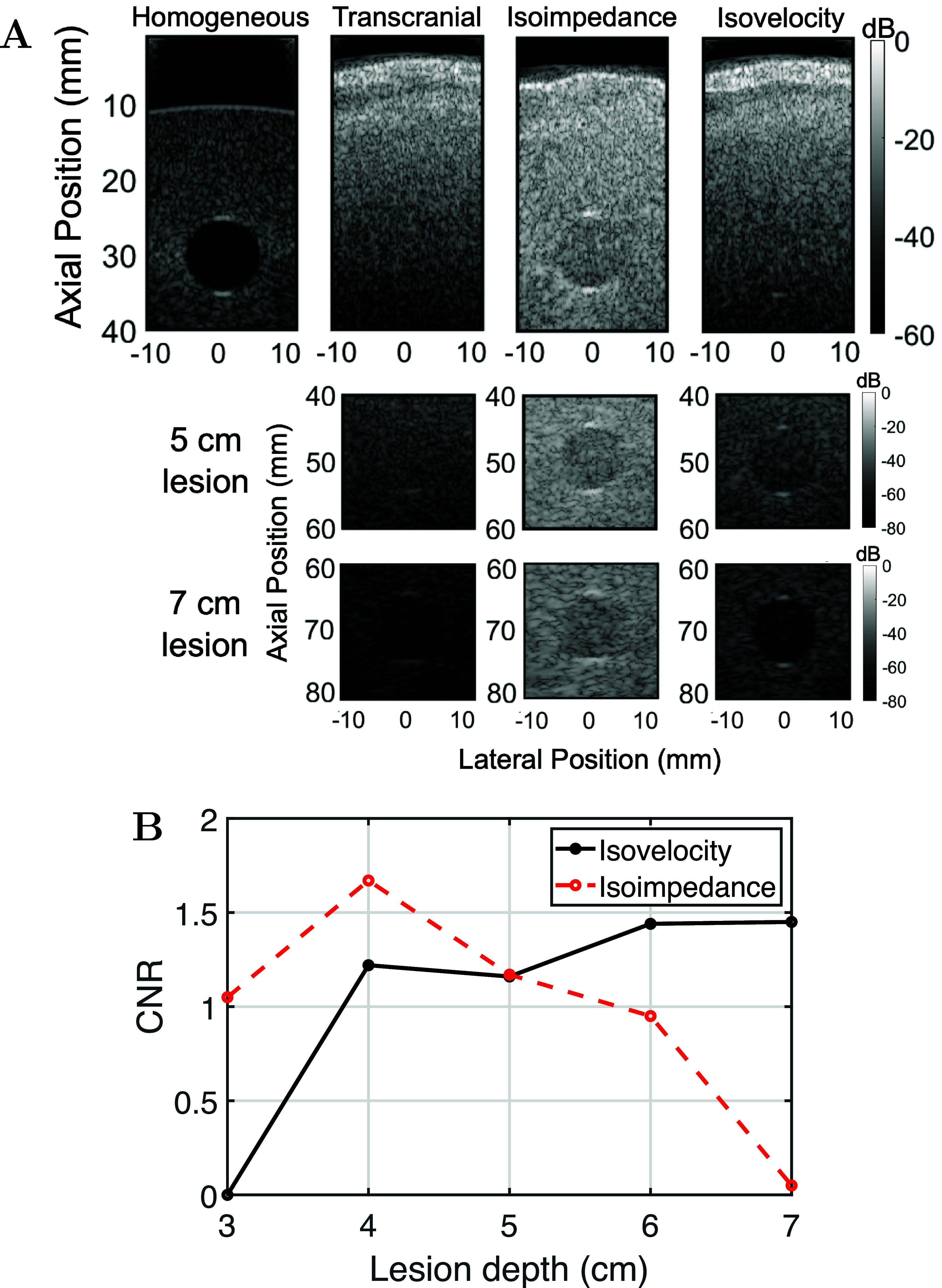
(A), Anechoic lesions with a 1 cm diameter at different shown at different imaging depths for each row (3, 5 and 7 cm) and for a different imaging scenarios for each column, namely from left to right, for a homogeneous medium, transcranially, transcranially but with the effects of multiple reverberations removed, and transcranially but with phase aberration removed. (B), Trend of CNR values for the isoimpedance and isovelocity cases as a function of imaging depth.

Figure 13(A), shows the B-mode images of the anechoic lesions for 4 different imaging scenarios and at 5 different depths *in silico*. The homogeneous case, without the human skull, is used a reference. The lesion in this reference is at 3 cm, a depth where the effects of reverberation are high. Then, the human skull was inserted under the transducer in the transcranial case. The effects of multiple reverberations are removed in the isoimpedance case and the effects of aberration are removed in the isovelocity case. To compare the relative impact of reverberation and aberration at difference depths, the isovelocity, isoimpedance, and transcranial simulations were performed for the lesion situated at 3, 4, 5, 6 and 7 cm of depth (figure 13(A)). It appears that reverberations are the dominant source of image degradation at least up to 5 cm of depth, with phase aberration improving image quality mainly after the 5 cm mark. In the transcranial case, where both mechanisms are present, the lesion is not clearly discernible until 7 cm of depth, where reverberations are substantially lower and phase aberration is virtually the only source of image degradation.

CNR measurements indicate the lesion image quality in the B-mode, and by extension the relative contribution of reverberation and phase aberration, when comparing isoimpedance and isovelocity B-modes. Figure 13(B) shows the CNR values for the isovelocity and isoimpedance B-modes as a function of lesion depth. The trend of the curves can be used to evaluate how CNR behaves as depth increases, with isovelocity values increasing and isoimpedance values decreasing with increasing depth. In figure 13(B), the qualitative observation that phase aberration and reverberations start to equally contribute to image degradation around 5 cm of depth is corroborated. Past that depth, removing aberration appears to have a higher impact on image quality, whereas at shallower depths, multiple reverberations are the main degrading mechanism, something that is also shown in the equivalent B-mode images in figure 13(A). The CNR peaks at 4 cm for the isoimpedance case and 7 cm for the isovelocity case. When comparing the lesion cases at 4 and 6 cm, the relative CNR decreased by 0.7 for the isoimpedance case and increased by 0.3 for the isovelocity case. At 5 cm, when the CNR for the two cases is the same, the CNR is 0.6 lower than isoimpedance case with the highest CNR and 0.3 lower than the isovelocity case with the highest CNR.

## 4. Discussion and conclusion

Previous studies have shown the validity of using simulations to isolate different sources of image degradation with respect to lung imaging (Soulioti *et al* 2021). Here, the effects of aberration and reverberation as the relate specifically to transcranial imaging are explored. It is shown that both aberration and reverberation affect image quality, even though most transcranial imaging methods are only focused on correcting for aberration caused by the skull.

Reverberation was shown to be both depth and target brightness dependent. Since reverberation is caused by multiply-reflected sound, shallower targets see more degradation due to the proximity to the skull and transducer, where sound waves are experiencing multiple reflections due to the mismatch in impedance values in the skull. Reverberation has less impact at deeper depths due to attenuation of the signal. Since this produces a haze around the image, brighter targets are less affected than less bright ones. Isoimpedance maps were used to investigate the effects of removing reverberation clutter on transcranial images so that aberration remains. The isoimpedance simulations were performed using clutter subtraction instead of isoimpedance acoustic maps, allowing for reverberation clutter to be removed and aberration to remain. However, using this method, trailing clutter, caused by distortions of the ballistic pulse, is not subtracted out, and remains in the images. This isoimpedance method, however, has previously been shown to be accurate up to −43 dB (Soulioti *et al* 2021). Aberration degrades the phase of the wave, causing errors in the PSF; however, when caused by the skull, it is both depth and target brightness independent. Aberration is an important factor for transcranial ultrasound, especially for therapy applications that only rely on the one-way propagation of sound to focus ultrasound to specific brain regions. However, for diagnostic applications, the two-way sound propagation required to transmit and receive sound introduces large impacts due to reverberation, especially in shallow, dim targets, where reverberation acts over a much larger area of the PSF than aberration.

All transcranial simulations were performed using a slice or volume of the temporal bone that was selected by hand. This is similar, however, to the way that sonographers would search for an ideal imaging window. We found that the microstructure of bone has little effect on the reverberation caused by the skull in our temporal bone case, where there is not much microstructure present. However, only a few skull samples were used. Previous studies have shown that the error in simulations increases due to skull homogenization in areas with more microstructure and porosity (Robertson *et al* 2018). It is also shown that the error increases with voxel size across all skull regions, but the error rises faster with increased voxel sizes for parietal and frontal bone compared to temporal bone (Robertson *et al* 2018). There is also an increased effect of aberration with skull thickness (Riis *et al* 2022). However, the impacts of lower resolution may be more consequential for other brain regions like the frontal and parietal regions. Future studies could include more skull samples and more skull regions of varying thicknesses and porosities. At our resolution of 0.105 mm, the error in the temporal bone was low but the error in the frontal and parietal regions was much higher. This means that, in this case where the temporal bone was used, simulations can be performed using the CT scans from clinical scanners. This study used synchrotron data to act as a high resolution ground truth compared to downsampled synchrotron data to act as the homogenized skull scan to allow for a direct comparison between skull scans with and without the microstructure. However, synchrotron data has a narrower energy band than clinical CT scanners, meaning that certain CT effects are not well represented in the downsampled synchrotron case. Future studies with more representative CT comparisons are needed for broader comparisons between the use of different imaging modalities in acoustic map conversions There is a slight difference before 15 mm in depth between the skull and simulation cases for the skull calibration. This is likely due to differences between the two. The simulation was run as closely as possible to the experimental case, but there are likely differences in the exact positioning of the skull and reflections from the water bath, leading to slight differences in the clutter pattern.

This study included three different frequencies. The skull homogenization was performed at 1 MHz to match previously performed attenuation calibrations (Pinton *et al* 2011a). This is a frequency that has overlapping relevance between imaging and therapy. The point target simulations were performed at 1.25 MHz in 2D and 3D to match the frequency of the 3D transducer modeled. The skull calibration and anechoic lesion simulations were performed at 2.5 MHz since the corresponding experimental results used that frequency. While different frequencies were used here to simulate specific transducers, these frequencies are all applicable to transcranial imaging. The experiments and corresponding simulations were performed using the P4–1 imaging transducer at 2.5 MHz. The 3D simulations were performed using a sparse matrix array specifically designed for human transcranial imaging, emitting at 1.25 MHz (McCall *et al* 2023), with the 2D point target simulations using the same frequency to match. However, the difference in simulation conditions due to the dense vs sparse geometry and lower frequency could result in differing levels of aberration and reverberation. Future studies could do a broader comparison of the relative contribution of reverberation and aberration across frequencies.

The PSF was used to analyze the impacts of reverberation and aberration on imaging brightness. From the PSF analysis, it is shown that the PSF to clutter ratio can decrease by over 0.3 when the target brightness is reduced due to reverberation. This value remains constant in the presence of only aberration. The PSF analysis alone does not allow for conclusions to be drawn on the relative contribution of phase aberration and reverberation. As shown in figure 8, the contribution of reverberation in the final image is highly dependent on target brightness, so even at a single imaging depth, conclusions can only be drawn for a certain reflectivity. Decreasing target brightness considerably affects image quality and might render the target indiscernible independently of phase aberration. We are only able to monitor changes in aberration strength or reverb strength relative to themselves. To account for this, reverberation and aberration were also compared on an anechoic lesion, allowing for depth comparisons without differences in reflectivity. In this case, it is also shown that the brightness of the preceding regions can increase by 30 dB due to reverberation when moving a target from 6 to 3 cm, while no variation is seen between these depths when just aberration is present. In addition, reverberation is the dominant source of degradation at shallower lesion depths of 3 and 4 cm, whereas aberration is the dominant source at deeper depths of 6 and 7 cm, where the maximum CNR occurs at 4 cm for the isoimpedance case and 7 cm for the isovelocity case. The minimum CNR occurs at 3 cm and 7 cm for the isovelocity and isoimpedance cases, respectively. In the lesion images, the scattering configuration is the same for the heterogeneous and lesion cases; however, the speckle brightness is depth dependent. Once the speckle is subtracted from the images, the image appears brighter. This is due to the increased contrast due to the removal of the reverberation clutter followed by normalization of the image. In all B-modes, images are displayed is dB relative to the maximum of each simulation. This could artificially decrease the visual effect of clutter in images. However, this scaling does not affect the CNR.

In addition to depth and brightness, coherence was investigated. It was found that in an anechoic lesion at 3 cm of depth, the removal of reverberation improves tissue coherence, particularly in short lags where the coherence value improves by over 0.3. When just aberration is removed, there is little difference in tissue coherence when compared to the transcranial case.

Lastly, the effect of harmonic imaging on reverberation clutter was investigated. It was found that harmonic imaging reduces the width of the main lobe for both homogeneous and transcranial cases, with decreases in reverberation clutter of 15 dB.

These simulations did not include shear wave or mode conversion modeling. In this study, the sound is focused at deep depths below the skull, leading to low angles of incidence with the skull. Shear waves and mode conversion are known to have a small impact at low angles of incidence (Clement *et al* 2004, Robertson *et al* 2017). However, the inclusion of these effects could change the modeling of reverberation and aberration. Future studies investigating aberration and reverberation with increased angles of incidence with the skull could provide a broader picture of how the skull impacts images, especially at shallow focal depths.

There are many different phase aberration techniques to improve ultrasound image quality (Flax and O’Donnell 1988, Jaeger *et al* 2015, Sharifzadeh *et al* 2020, Ali *et al* 2022); however, there are fewer reverberation suppression techniques (Byram *et al* 2015, Shin *et al* 2016, Brickson *et al* 2021). Since these effects result from different mechanisms of PSF degradation, they typically need different correction techniques. However, in a linear system, these correction techniques should be separable and multiple correction techniques could be applied. There is, however, one recent technique that both corrects aberration and suppresses reverberation clutter (Zhuang *et al* 2024). Due to the impact of reverberation on image quality, more research into reverberation suppression and combined reverberation and aberration approaches are required.

In conclusion, reverberation clutter significantly degrades ultrasound imaging in the scattering environment of the human brain compared to phase aberration, especially at depths shallower than 5 cm. The presented results suggest that clutter reduction methods, such as harmonic imaging or spatial coherence imaging, may contribute significantly to image quality improvements.

## Data Availability

All data that support the findings of this study are included within the article (and any supplementary information files). Data will be available from 2025 October 28.
